# Longitudinal outcome prediction of prostate cancer patients on active surveillance using multiple instance learning

**DOI:** 10.1117/1.JMI.12.6.061408

**Published:** 2025-10-14

**Authors:** Filip Winzell, Ida Arvidsson, Kalle Åström, Niels Christian Overgaard, Felicia-Elena Marginean, Athanasios Simoulis, Anders Bjartell, Agnieszka Krzyzanowska, Anders Heyden

**Affiliations:** a Lund University, Centre for Mathematical Sciences, Lund, Sweden; b Skåne University Hospital, Malmö, Sweden; c Lund University, Division of Urological Cancers, Dept. of Translational Medicine, Lund, Sweden

**Keywords:** digital pathology, prostate cancer, artificial intelligence, deep learning, whole slide images, multiple instance learning, patient outcome

## Abstract

**Purpose:**

To avoid over-treatment of prostate cancer patients following screening for elevated prostate-specific antigen levels, keeping patients on active surveillance has been suggested as an alternative to radical treatment. This means recurring visits for patients with low-grade cancer to monitor progression. Our aim was to develop an artificial intelligence-based model that can identify high-risk patients in a cohort of prostate cancer patients on active surveillance.

**Approach:**

We have developed a multiple instance learning-based framework for predicting the longitudinal outcomes for prostate cancer patients on active surveillance. Our models were trained only on whole-slide images with patient-level labels without using explicit Gleason grades. We employed the UNI-2 foundation model and the well-established attention-based multiple instance learning approach. We further evaluated our models by fitting Cox proportional hazards models and testing them on an external dataset.

**Results:**

With this approach, we achieved an average area under the receiver operator characteristic curve of 0.958 (95% CI, 0.957 to 0.959). Fitting Cox models to the predicted probabilities achieved a C-index of 0.824 and a hazard ratio of 2.32. However, all models showed a large drop in performance when evaluated on an external dataset.

**Conclusion:**

We show that avoiding Gleason grades is beneficial for longitudinal outcome prediction of prostate cancer. Our results suggest that benign prostate tissue contains prognostic information. However, before our models could be used clinically, much more work remains to improve the generalization.

## 1 Introduction

Prostate cancer is the world’s most common male cancer diagnosis, with more than 1.4 million reported new cases in 2020,^1^ and it is expected to rise towards 3 million by 2040.^2^ This poses a requirement for early detection to limit the loss of life and future healthcare costs in a system already under pressure. Screening for elevated levels of prostate-specific antigen (PSA) has been shown to significantly reduce mortality rates.^3^^–^^5^ However, there is evidence of such screenings leading to an unacceptable increase in overdiagnosis and subsequent over-treatment.^4^^,^^6^^,^^7^ To mitigate this, active surveillance has emerged as an alternative to the radical treatment following a positive screening result, where low-risk patients are monitored with recurring visits. Thereby, treatment of harmless tumors can be avoided while still enabling detection of prostate cancer cases at an earlier stage.^6^^,^^8^ However, continuous monitoring of patients will increase the workload of the pathologists and require patients to undergo the prostate biopsy procedure more often. With the tremendous rise of research in computational pathology over recent years, an AI solution able to predict whether to keep patients on active surveillance or when they will require further treatment would be highly valuable in this scenario.

In today’s clinical practice, prostate biopsies are graded according to the Gleason grading system. Because of its latest revision by the International Society of Urological Pathology (ISUP), the grading system now consists of four grades of histological patterns: benign and grades 3 to 5.^9^ These are then summed into ISUP grade groups, ranging from 1 to 5, where 1 corresponds to the lowest grade of prostate cancer (i.e., not including benign).^10^ The assigned ISUP grade groups have a large impact on prognosis and choice of treatment; however, due to the subjectivity of the grading system, there are concerns regarding intra- and inter-observer variability.^11^ In addition, given that each diagnosis requires ∼8 to 12 biopsy cores, each generating a gigapixel whole-slide image (WSI), determining a diagnosis is tedious for pathologists. The development of automated Gleason grading algorithms based on deep learning has the potential to dramatically reduce this workload.^12^^–^^15^ However, these algorithms are still intrinsically limited by the variability of the Gleason grading system.

Hence, to both overcome the issues regarding the variability and uncertainty of the Gleason grading system and to make longitudinal predictions on patients on active surveillance, we have developed an end-to-end framework for predicting the need for radical treatment of patients on active surveillance. Our models are trained on a dataset collected within the Prostate Cancer Research International Active Surveillance (PRIAS) study, where the input is a set of prostate biopsy WSIs following a patient visit, and the output is the probability of requiring treatment. To avoid using explicit Gleason grades, we utilized the principles of attention-based deep multiple instance learning.^16^ We hypothesize that by training on patient outcomes, the model will be able to learn the tissues’ prognostic properties directly, thereby avoiding learning on noisy Gleason grades. In computational pathology, numerous algorithms for slide-level predictions have been developed in recent years.^13^^,^^17^^,^^18^ Nonetheless, in this work, we present a solution moving toward the next level, with patient-level predictions. We argue that this solution could assist pathologists in detecting high-risk patients and thereby mitigate the drawbacks of prostate cancer screening.

### 1.1 Related Work

The European Randomized Screening for Prostate Cancer (ERSPC) trial was initiated in 1992 to investigate whether prostate cancer mortality could be reduced by PSA screening.^19^ Early studies concluded that PSA screening reduces mortality; however, with estimated rates of over-treatment as high as 50% (defined as the amount of men with elevated PSA values who would not show any clinical symptoms in their lifetime).^19^ To improve the diagnostic methods in the screening program, enhanced blood tests^20^^,^^21^ and the use of MRI^22^ have been suggested, with promising results. Following a recent European Union recommendation, Bratt et al.^4^ evaluated the feasibility of organized prostate cancer screening in Sweden. They found that with the use of MRI, a biopsy could be avoided for over half of the patients with elevated PSA values. Blood-based liquid biopsies have in recent years been suggested as a powerful tool for biomarker testing in prostate cancer.^21^ Fonseca et al.^20^ showed that the circulating tumor DNA fraction (ctDNA%) is a strong predictor of progression and survival of patients with metastatic castration-resistant prostate cancer. However, the value of including liquid biopsies in a screening program is yet unclear and warrants further investigation.^21^

To avoid the tedious process of acquiring instance-level labels, multiple instance learning (MIL)-based algorithms using slide-level labels have been researched extensively in computational pathology. Ilse et al.^16^ conducted experiments as part of their introduction of attention-based deep multiple instance learning (ABMIL), experiments on two smaller histopathology datasets from breast cancer and colon cancer patients, showing promising results. Campanella et al.^13^ used MIL and recurrent neural networks to classify WSIs from prostate cancer, basal cell carcinoma, and breast cancer patients, achieving an area under the curve (AUC) of 0.98 for all types. Zhu et al.^23^ used a convolutional autoencoder combined with an MIL approach to predict heart transplant rejection. To overcome issues with data efficiency, Lu et al.^17^ developed the framework *clustering-constrained attention multiple instance learning* (CLAM), for the classification of renal cell carcinoma, non-small-cell lung cancer, and the detection of lymph node metastasis. One of their main contributions is the extension of an ABMIL framework to a multi-class WSI classification. Yang et al.^24^ further built upon the CLAM framework by introducing *Intensive-Sampling MIL*. They used a two-branch system, where the second sample patches focused on regions of suspected lesions, achieving better AUC than previous methods (including CLAM) on several datasets. This has also been applied in similar ways to prostate cancer datasets. Mun et al.^18^ used an attention-based deep MIL approach for automatic Gleason grading, achieving higher performance than CLAM on their own datasets. However, they also experienced a drop in performance when tested externally, hypothetically, due to differences in stain color distribution between their datasets.

Furthermore, several studies have shown a strong prognostic potential of AI-predicted Gleason grades, using Cox regression models to estimate disease progression based on the predicted grades.^8^^,^^25^ Liu et al. developed a deep learning-based system for predicting prostate cancer on the patch level using only benign biopsy WSIs, with an AUC of 0.701 (95% CI, 0.700 to 0.701).^26^ They aggregated the predicted tile-level scores to slide-level and then to patient-level using the 75th percentile, and the median, respectively, achieving a patient-level AUC of 0.739 (95% CI, 0.682 to 0.796). Their primary finding is that it is possible to detect indications of prostate cancer in benign tissues using deep learning-based models. However, they did not train explicitly on patient outcomes, and only on benign tissue as defined by the Gleason grades. Pinckears et al.^27^ developed a deep learning-based biomarker for the prediction of biochemical recurrence of prostate cancer. They used an ImageNet pre-trained ResNet-50 for predicting years to recurrence based on prostatectomy tissue microarrays, together with a nested control study, achieving promising results. Chelebian et al.^28^ used a CLAM-based method with a ResNet18, pre-trained with contrastive learning, as a feature extractor to predict future detection of prostate cancer. They used a cohort of men with elevated PSA values and only slides originally classified as benign, where the positive patients received a prostate cancer diagnosis (any ISUP grade) within 30 months, and the negative patients remained cancer-free for 8 years. On slide-level labels, they achieved an AUC of 0.81, and by assigning patients with any predicted high-risk slides as positive, they achieved an AUC of 0.82 at the patient-level.

One of the most significant breakthroughs in computer vision in recent years has been the development of foundation models for powerful feature extraction.^29^^–^^31^ Following the success of these models, various attempts have been made to develop foundation models specifically for computational pathology. Wang et al.^32^ developed a self-supervised contrastive learning approach for training a general feature extractor for histopathology (CTransPath). Since then, numerous histopathological foundation models have followed.^33^^–^^36^ Notably, Chen et al.^37^ developed the vision transformer (ViT-l)-based model called UNI, pre-trained using the self-supervised learning approach DINOv2 on over 100k WSIs across more than 20 tissue types. In early 2025, the second version of UNI was released, which is the larger ViT-h, trained on more than 350k WSIs.

## 2 Method

### 2.1 Data

In this study, we utilized a cohort collected within the PRIAS study at three hospitals (Kristianstad, Lund, and Malmö) in Skåne County, Sweden. The inclusion criteria for the study can be summarized as men with histologically proven adenocarcinoma of the prostate who are fit enough for curative therapy. They could at most have two prostate cancer-positive biopsy cores, with at most ISUP grade group 2 (Gleason score 3 + 3 or 3 + 4) and at diagnosis, the PSA levels should be ≤10  ng/mL and PSA density <0.2.^8^ More details on the inclusion and exclusion criteria can be found at Ref. 38. Our cohort consists of 180 patients monitored from 2007 to 2020, hereafter referred to as the PRIAS cohort. After removing the patients who had discontinued the study due to some reason unrelated to prostate cancer, 145 patients with a total of 4704 WSIs remained. See Fig. 1 for the Consolidated Standards of Reporting Trials (CONSORT) chart. The slides were originally scanned at ×40 magnification (0.247  μm/pixel); however, we used images downsampled to ×10 magnification. Additional details on the PRIAS cohort are described in Arvidsson et al.^8^

**Fig. 1:**
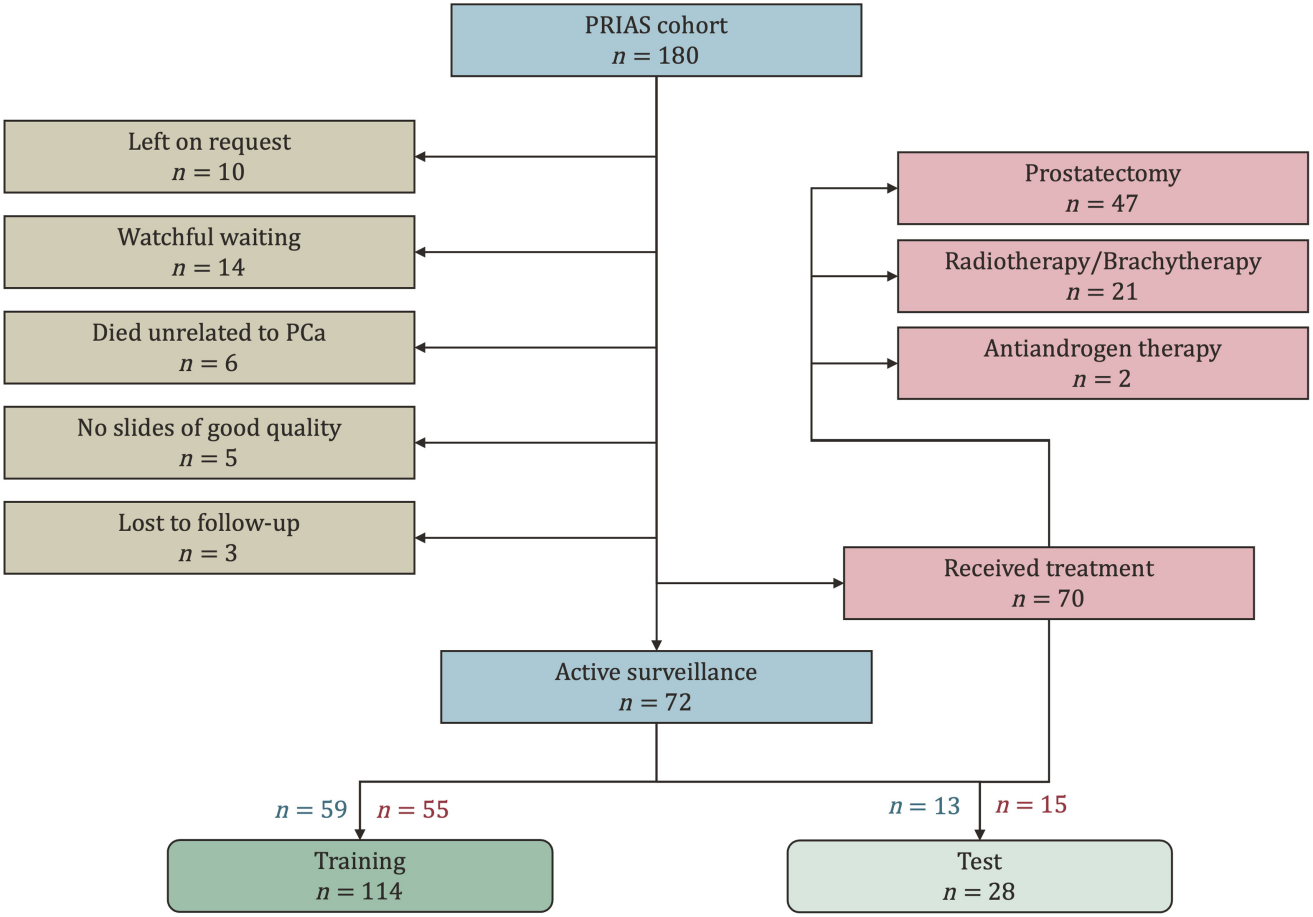
CONSORT diagram of the PRIAS cohort. The active surveillance group (n=72) and treated group (n=70) were randomly split into training and test with an 80/20% ratio.

The remaining patients have two possible outcomes: whether they remained on active surveillance throughout the study (0) or received treatment (1) for prostate cancer. Note that patients who did receive treatment were subsequently removed from the study, and do not have any additional follow-up visits, since they, from that point, are no longer on active surveillance. The cohort was randomly split into a training (80%, n=114) and test set (20%, n=28), keeping the ratios of labels similar. The models were trained to predict whether a patient on active surveillance should receive treatment (no = 0, yes = 1) given all WSIs from a single visit. For the treated group, we hypothesized that visits prior to 30 months before the treatment decision are too old for the model to capture any prognostic information in this limited dataset. Hence, we only kept visits within 30 months before the final visit for the treated group. For the active surveillance group, we do not know how the disease progressed after the final visit. Hence, we right-censored this data by removing the final visit. For example, suppose we have two patients with different treatment decisions. The patient who remained on active surveillance has four visits in the study, and thus, the three earlier visits are eligible to use and given the label 0. The patient who received treatment has had three visits, with the first one being more than 30 months before the last. Then, the first visit is subsequently removed, and the remaining two are given the label 1. Each of these five eligible visits is used separately in training (i.e., no information from prior visits is included in the prediction). This example of the labeling of the patients’ visits is illustrated in Fig. 2. In total, this gave us 300 remaining visits with 3085 WSIs divided into the training and test sets. Naturally, we split the dataset into test and train on the patient level to ensure that no patient has any visits in both splits. Slides from before 2011 were deemed to be of insufficient quality, mainly due to fading, and therefore removed in a refined version of the cohort. This reduced the number of visits to 269 with 2775 WSIs (three patients had no biopsies remaining). The PRIAS dataset split is summarized in Table 1.

**Fig. 2:**
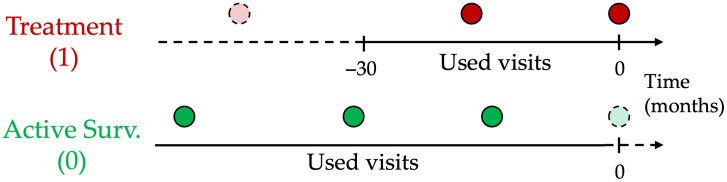
Illustration of the timelines of two patients within our cohort. For the patients who did receive treatment, the visits prior to 30 months before the final visit were removed. For the patients who remained on active surveillance, the final visit was removed.

**Table 1:** Summary of the PRIAS cohort train and test split and the external dataset. Roughly 50% of patients in the PRIAS cohort received treatment. We removed the final visit for the active patients and any visits prior to 30 months of the final visit for the treated group. This gave slightly more visits for the active group. We also present the refined version of the dataset without slides prior to 2011. On the external dataset, we removed all patients with ISUP grade 1, which reduced the percentage of positive patients.

	Patients	Active	Treated	Active visits	Treated visits	Total visits
Train	116	58	58	135 (56.5%)	104 (43.5%)	239 (2446 WSIs)
Test	29	14	15	31 (50.8%)	30 (49.2%)	61 (639 WSIs)
Refined—no slides prior to 2011						
Train	114	58	56	120 (56.5%)	97 (43.5%)	217 (2230 WSIs)
Test	28	13	15	25 (48.0%)	27 (52.0%)	52 (545 WSIs)
	Patients	Negative	Positive	Negative visits	Positive visits	Total visits
External	182	125	57	125 (68.7%)	57 (31.3%)	182 (450 WSIs)

In addition to this, we evaluated our methods on an external dataset of prostate biopsies with undetected prostate cancer collected at the University Hospital of Umeå, Sweden.^39^ This dataset consists of 213 patients who underwent one visit with a PSA test and prostate biopsies. This amounted to 587 H&E-stained WSIs, which were all initially diagnosed as benign. Each patient is given the label benign if they continued to be prostate cancer-free after 8 years following this biopsy. If they instead exhibited any ISUP grade in a subsequent re-biopsy within 30 months, they are labeled as undetected prostate cancer cases. In total, 125 patients (with 333 WSIs) remained cancer-free, and 88 patients (with 254 WSIs) were diagnosed with prostate cancer. According to the PRIAS protocol, patients with ISUP grade 1 will likely be regarded as low-risk and be kept on active surveillance. However, this is not always the case; if, for example, a patient has an increasing number of positive biopsies, they might receive treatment. Due to this ambiguity, we removed all 31 patients with ISUP grade 1 from the external dataset, reducing the number of positive patients to 57 (with 117 WSIs). See Ref. 39 for more details on this dataset.

### 2.2 Attention-Based Deep Multiple Instance Learning

Inspired by CLAM,^17^ the proposed method employs ABMIL for patient-level classification. To train on patient outcomes, the framework is extended from classifying single WSIs to classifying a set of WSIs. The model is divided into two stages: (1) feature extraction and (2) MIL-based outcome prediction (see Fig. 3). The first stage begins with automatic segmentation of each of the input WSIs into foreground and background pixels, using Otsu’s method.^40^ The segmented foreground area, i.e., all pixels containing tissue, is then divided into 280×280 patches, with a 75-pixel overlap. Each patch is subsequently transformed into latent representations using the UNI-2 foundation model,^37^ which in its default setting generates vectors of dimension 1×1536.

**Fig. 3:**
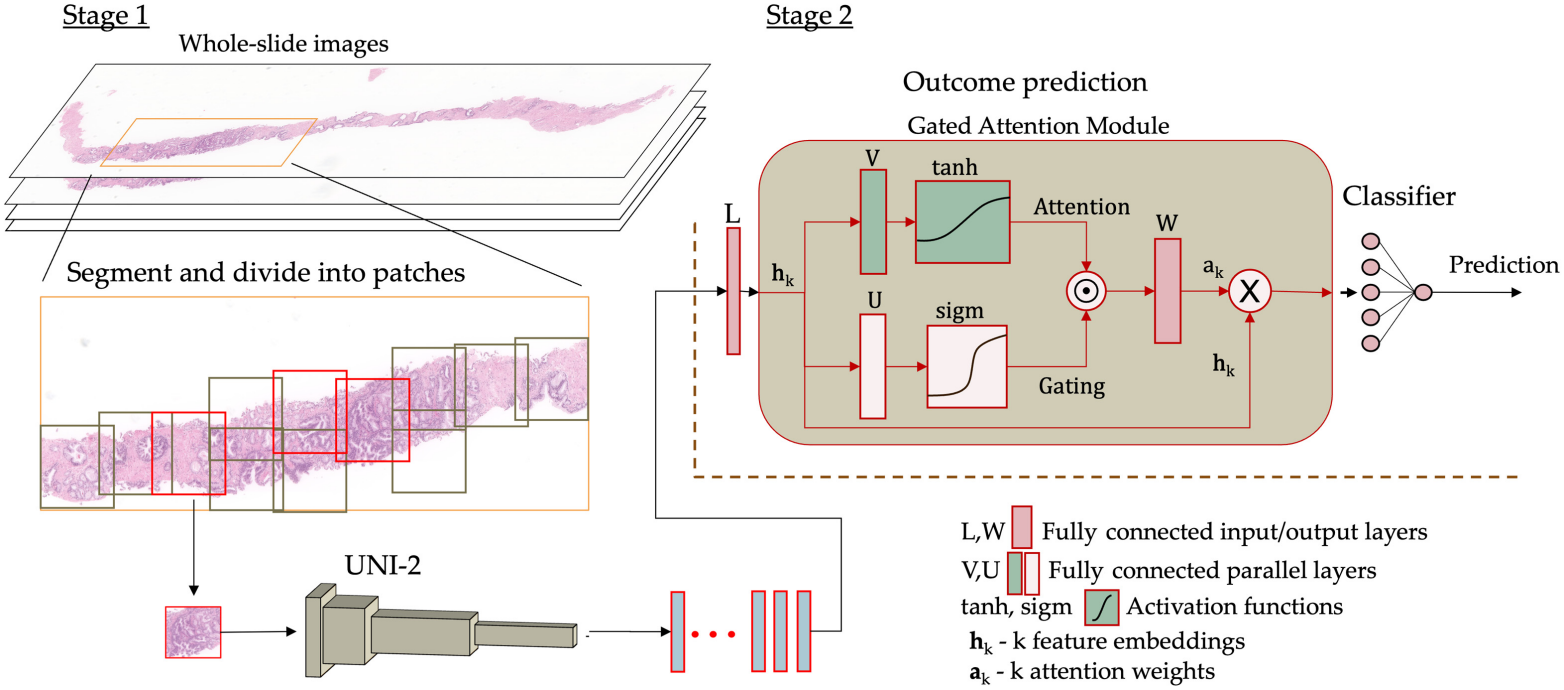
Illustration of the framework. In the first stage, the WSIs are segmented automatically using Otsu’s method into foreground, containing all tissue, and background pixels, and then divided into patches. The UNI-2 foundational model is used to transform the patches into latent representations. In the second stage, the k feature vectors are processed with a fully connected input layer L, into embeddings $h_{k}$, followed by two pathways with parallel fully connected layers, V and U. The attention pathway uses a tanh(·) activation function, allowing both negative and positive outputs. The second pathway adds a gating signal with a sigmoid(·) activation, bounded between [0, 1]. The final attention weight $a_{k}$ is computed by the fully-connected layer W, following an element-wise multiplication of the two signals. Finally, the computed attention weight $a_{k}$ and the corresponding embeddings $h_{k}$ are multiplied, and a one-layer MLP makes the outcome prediction as a probability.

Hence, the input to the second-stage ABMIL module is a number of k feature vectors extracted from k patches of the input WSIs (i.e., a matrix $I\in R^{k\times 1536}$). Note that this k will vary for each patient visit. During training, we only update the weights of the second-stage module, which is trained from scratch. Under the MIL problem formulation, we consider the feature vectors as the bag of instances with the binary patient outcome as the label (i.e., active surveillance, 0, or received treatment, 1). We use the gated attention mechanism as suggested by Ilse et al.,^16^ with a fully connected input layer and a fully connected classifier at the end.

Despite its popularity, concerns have been raised that ABMIL tends to concentrate attention on a small number of instances, leading to overfitting and suboptimal performance.^41^^,^^42^ A possible way to mitigate this is to maximize the entropy of the learned attention weights during training.^41^ Similarly, we introduce a negative entropy term to our loss function as

$$L_{\mathrm{aem}}=-H(a)=\underset{a_{k}\in a}{\sum }a_{k}\;\log\;a_{k},$$ (1)

where a is the learned attention weights for one bag. As suggested by Ref. 41, we combine this loss with the binary cross-entropy loss ($L_{\mathrm{bce}}$) using a tunable hyperparameter λ into the total loss

$$L=L_{\mathrm{bce}}+\lambda L_{\mathrm{aem}}.$$ (2)

Another way to address the overfitting issue of ABMIL is to stochastically sample k instances (SKS), as suggested by Zhang et al.^42^ However, unlike their approach, we do not consider the top-k attention weights but simply sample instances before computing the attention weights. In addition to this, we also compare this with our previous approach, where we selected the top 256 instances based on predicted probabilities of containing malignant tissue.^43^ Here, we trained a separate DenseNet201 on the task of separating patches into benign or malignant (Gleason grade 3 to 5). Hereafter, we refer to the DenseNet model as GG-Net and this approach as the GG-model. For more details, see the original publication.^43^

### 2.3 Training and Evaluation

We train our models with six-fold cross-validation, each for 100 epochs with early-stopping using the Adam optimizer with decoupled weight decay regularization.^44^ On each fold, we select the best model based on the highest achieved AUC on the validation fold. We augment our dataset by applying random HSV color transformations to our image patches. Furthermore, we randomly apply Gaussian noise and dropout to the feature vectors computed by UNI-2. We evaluated our models as an ensemble with the area under the receiver operating characteristic curve (AUROC) by averaging the predicted probabilities over the six models. We constructed the ROC curves and subsequently calculated the AUC using the R package “pROC.” All results are presented as averages, with 95% confidence intervals (95% CI) by bootstrapping the test dataset at the patient-level. To evaluate statistical significance between the models, we performed a pairwise analysis of the bootstrap confidence intervals by computing the difference in metric for each resampled dataset. If the 95% CI included zero, the difference was considered insignificant. Moreover, we analyzed the accuracy, sensitivity, and specificity of our models in a hypothetical clinical setting. To select an operating point, we constructed ROC curves of the average probabilities on the whole training data (including the validation fold) and selected the classification threshold that maximized Youden’s J statistic, defined as J=Sensitivity−Specificity−1.^45^ Maximizing this means maximizing the height from the random decision line. We then kept this threshold when evaluating on the test and external datasets. Our code for training and evaluating our models will be openly accessible at https://github.com/fwinzell/PRIAS_outcome_prediction.

### 2.4 Cox Regression Models

To further analyze the predictive power of our models, we fit time-dependent Cox proportional hazards regression models with the predicted probability of treatment as a single covariate. Here, we divide the patients into an active surveillance group and a treated group. Given that we have several visits for each patient, and visit-level predictions with time-to-event data (time to treatment or censoring), it is appropriate to fit a time-dependent Cox regression model, with the predicted probability as a single time-dependent covariate. For patient i at a time t, the hazard function is modeled as

hi(t)=h0(t)·exp(β·y^i(t)), (3)

where $h_{0}(t)$ is the baseline hazard, ${\hat{y}}_{i}(t)$ is the predicted probability of treatment, and β is the fitted regression coefficient. Here, the time is defined as months since the first baseline visit. We fit the Cox models using the “coxph” function from the R package “survival.” To measure the predictive power of our fitted models, we use the hazard ratio, defined as the factor by which the hazard changes when the covariate is increased by one unit. Because we have probabilities ranging from 0 to 1 as our covariate, we set the unit increase to 0.1, i.e., HR=exp(β·0.1). To evaluate whether the association between the predicted probability ${\hat{y}}_{i}(t)$ and risk of treatment (hazard) is statistically significant, we use Wald’s test. It tests the null hypothesis that β=0, i.e., the covariate has no effect. Furthermore, the concordance index (C-index) is used to measure the predictive performance of the models. It is a measure of how well the model orders the time of events in the data, where 1.0 is a perfect score and 0.5 corresponds with random guessing.

## 3 Results

Our basic model is trained on all instances using standard binary cross-entropy, with only learned attention weights to aggregate features. In addition to this, we evaluate models trained with attention entropy maximization (AEM), stochastic sampling of instances (SKS), and with our malignancy predictions as a feature selector (GG). We hypothesized that keeping the older, faint slides from before 2011 would improve the generalization of our models; hence, these models were trained on the full dataset. However, given our previous findings,^43^ we trained the GG model on the refined cohort. For comparison, all models were evaluated on the full test set. The results are summarized in Table 2 and Fig. 4. The basic model outperformed the other models on both the test set (AUROC, 0.958; 95% CI, 0.957 to 0.959) and the external dataset (AUROC, 0.699; 95% CI, 0.697 to 0.702). We evaluated the statistical significance of these results with a pairwise 95% confidence interval of difference test. Here, only the difference between the basic model and the GG and AEM models on the test set was significant (see Fig. 5). It should be noted that with Bonferroni correction for multiple comparisons, there are no statistically significant differences. We selected operating points for our models based on the maximum Youden’s J statistic. These are presented in Table 3, together with total accuracy, sensitivity, and specificity on both the test and external datasets. On the test dataset, the basic model performed the best with an accuracy of 0.871 (95% CI, 0.868 to 0.873) with an even balance between sensitivity (0.835, 95% CI, 0.831 to 0.840) and specificity (0.905, 95% CI, 0.902 to 0.908). However, on the external, the AEM model reached the highest accuracy (0.683, 95% CI, 0.681 to 0.685), albeit with a low sensitivity of only 0.263 (95% CI, 0.260 to 0.267). Notably, the GG model selected the smallest operating point of 0.28 and subsequently scored a high sensitivity (0.909, 95% CI, 0.907 to 0.912) and a low specificity (0.209, 95% CI, 0.207 to 0.212), indicating that the selected operating points were sub-optimal for these models.

**Table 2:** Summary of the models’ performances on the test and external datasets in AUROC. The test set was bootstrapped (n=1000) at the patient-level to estimate variance. The results are presented as an average metric (95% CI). The bold numbers indicate the best average performance. The right-most column shows the change in mean AUROC between the test and external datasets.

Model variations	AUROC (test)	AUROC (external)	Mean diff.
Basic	**0.958** (0.957 to 0.959)	**0.699** (0.696 to 0.702)	↓ 0.259 (27.0%)
SKS	0.906 (0.903 to 0.908)	0.677 (0.674 to 0.680)	↓ 0.229 (25.2%)
AEM	0.846 (0.843 to 0.849)	0.611 (0.608 to 0.614)	↓ 0.235 (27.8%)
GG	0.855 (0.852 to 0.858)	0.678 (0.675 to 0.681)	↓ 0.177 (20.7%)

Note: bold values indicates the best performance.

**Fig. 4:**
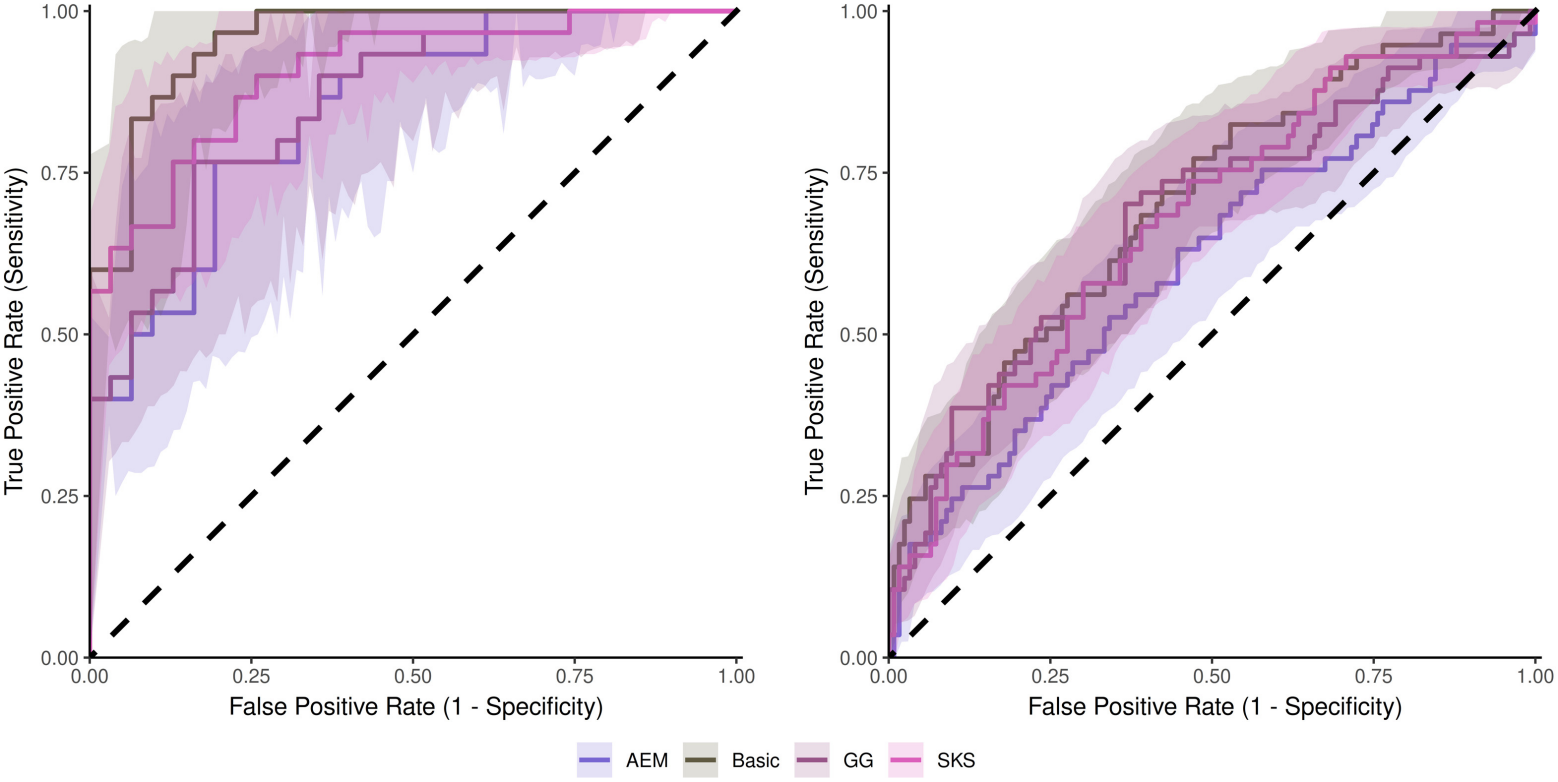
ROC curves with 95% confidence intervals, estimated by bootstrapping the PRIAS test dataset (left) and external dataset (right) on patient-level (n=1000).

**Fig. 5:**
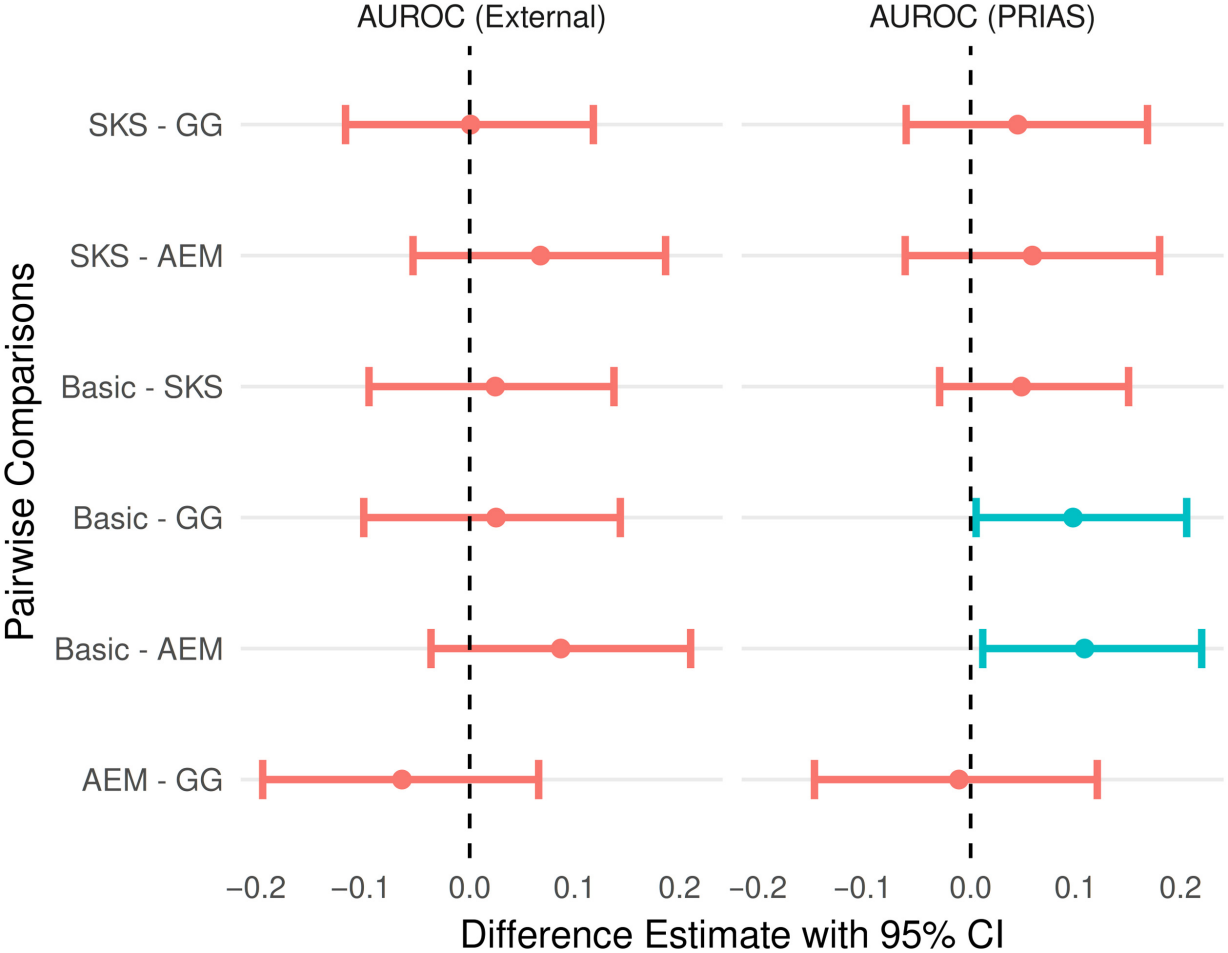
Forest plot showing the results from the pairwise confidence intervals of the difference test. Here, each pair of models is evaluated against each other, and the difference is considered significant if the 95% CI does not overlap zero. Red color indicates a non-significant difference, and blue indicates significance.

**Table 3:** Total accuracy, sensitivity, and specificity of the models, using the operating points that maximized Youden’s J statistic on the training data. Evaluated on the test and external datasets. The results are presented as averages and 95% CIs after bootstrapping (n=1000) the datasets.

Model variations	Op. point	Accuracy	Sensitivity	Specificity
Test				
Basic	0.41	**0.871** (0.868 to 0.873)	0.835 (0.831 to 0.840)	0.905 (0.902 to 0.908)
SKS	0.53	0.804 (0.801 to 0.807)	0.800 (0.795 to 0.804)	0.809 (0.804 to 0.813)
AEM	0.42	0.708 (0.704 to 0.711)	0.606 (0.601 to 0.612)	0.806 (0.801 to 0.810)
GG	0.28	0.803 (0.800 to 0.806)	0.766 (0.761 to 0.771)	0.839 (0.835 to 0.843)
External				
Basic	0.41	0.633 (0.631 to 0.635)	0.578 (0.574 to 0.582)	0.659 (0.656 to 0.661)
SKS	0.53	0.651 (0.648 to 0.653)	0.474 (0.470 to 0.478)	0.732 (0.730 to 0.735)
AEM	0.42	**0.683** (0.681 to 0.685)	0.263 (0.260 to 0.267)	0.877 (0.875 to 0.878)
GG	0.28	0.431 (0.429 to 0.434)	0.909 (0.907 to 0.912)	0.209 (0.207 to 0.212)

To add a layer of explainability to our results, we visualized the attention weights in Fig. 6 by constructing heatmaps over the WSIs from a patient visit. The attention weights were normalized into quartiles and projected onto each corresponding image patch. Additional heatmaps from multiple patients can be found at https://github.com/fwinzell/PRIAS_outcome_prediction.

**Fig. 6:**
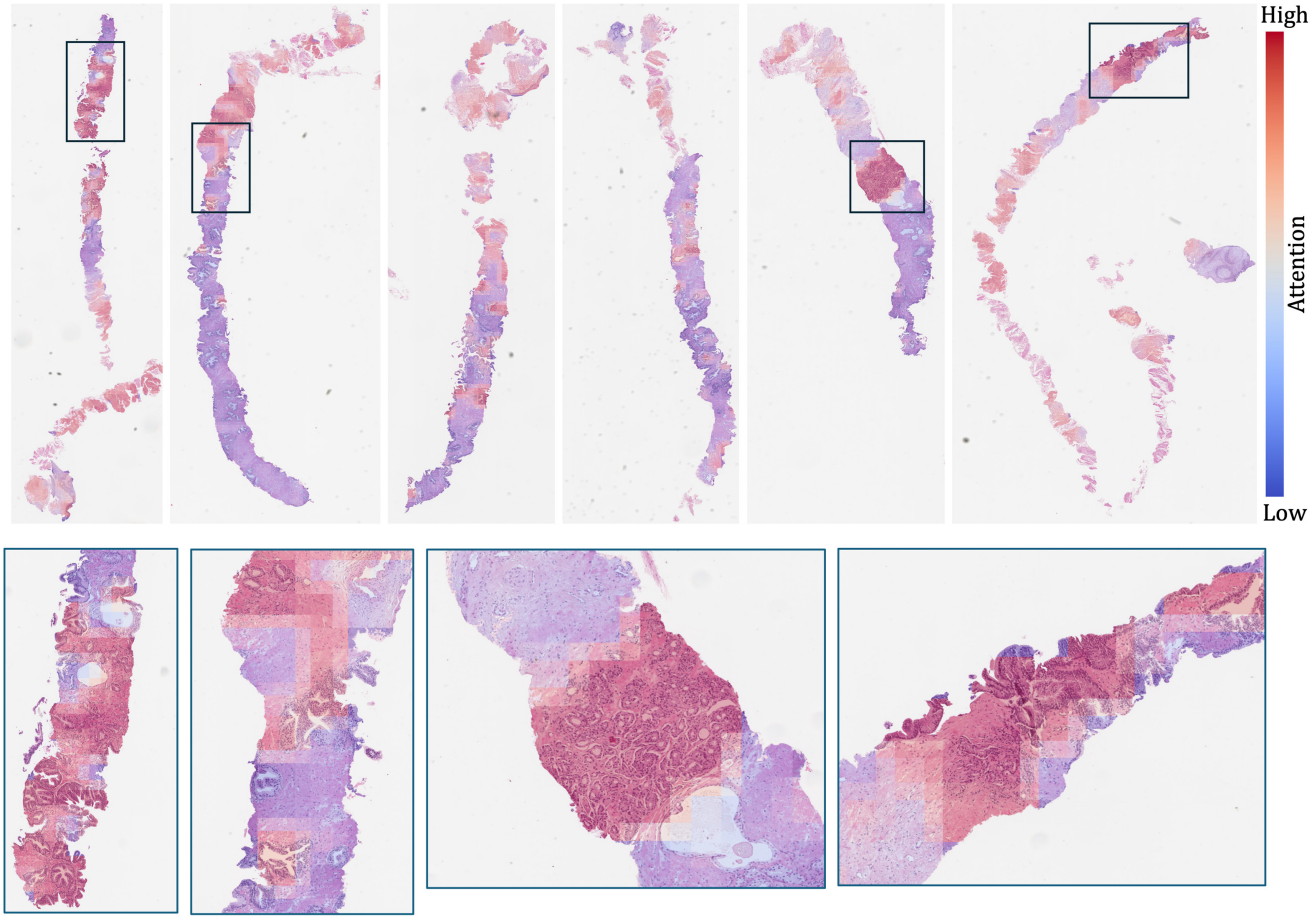
Heatmap of attention across one patient visit from the test dataset using the basic model. Red color means higher attention weights, and blue color means less attention. For regions where patches overlap, the average attention is projected. This patient was correctly classified as Treatment.

We evaluated the predicted probabilities on the test set with time-dependent Cox proportional hazards models. For each of our four models, we fitted a Cox model with the predicted probabilities as a time-dependent covariate. Results from fitting these models can be found in Table 4. We defined the unit increase in the probability as a 0.1 increase to make the hazards ratio more interpretable. The basic model gave the best fit, with a C-index of 0.824 and a hazards ratio of 2.32 (95% CI, 1.32 to 4.09) (i.e., an increase in predicted probability of 0.1, by this model increases the risk of treatment by a factor of 2.32). The basic and SKS models displayed significant effects of the predicted probabilities following Wald’s test at a 95% confidence level. We also fitted Cox regression models to the predicted probabilities on the external data (Table 5). Because we here only have one prediction (and time point) per patient, we used a time-independent model. Analogously to the AUROC, the basic model gave the highest C-index of 0.699. However, the GG model produced the highest hazard ratio of 1.45 (95% CI, 1.21 to 1.73).

**Table 4:** Results from fitting time-dependent Cox proportional hazards models on the test dataset. The hazard ratio is the change in hazard when the estimated probability is increased by 0.1. We also present the C-index and the p values following Wald’s test.

Model variations	Hazard ratio	C-index	Wald’s test (p)
Basic	2.32 (1.32 to 4.09)	0.824	0.003^a^
SKS	1.42 (1.10 to 1.83)	0.780	0.007^a^
AEM	1.22 (0.98 to 1.51)	0.714	0.076
GG	1.33 (0.99 to 1.80)	0.736	0.062

a Indicates statistical significance with a p value below 0.05.

## 4 Discussion

The basic model significantly outperformed the GG model on the test set (without multiple comparisons correction), suggesting that ABMIL on all instances has an advantage over using traditional Gleason grades for outcome prediction of prostate cancer patients. This is supported by other studies, suggesting that benign prostate tissue does contain relevant prognostic information.^26^^,^^28^ We did experience a large drop in performance for all models on the external dataset. There are some notable differences between these datasets, including that the external dataset does not follow patients on active surveillance and that the labels are not defined based on treatment decisions, but rather any occurrence of prostate cancer as defined by the Gleason grades. This might also explain why the GG model had the smallest drop in average AUROC of all models (see Table 2). Furthermore, the external dataset contains slides collected between 1997 and 2016, and we have not assessed the quality of the older slides compared with the PRIAS dataset. Although these differences can explain the difficulties in getting the models to generalize, one would expect a well-fitted model to perform better. Moreover, given that there are no statistically significant differences between the models’ performance on the external dataset, we cannot draw any definite conclusions on which model generalized the best. Regardless, the AEM and SKS variations were introduced to avoid overfitting and improve generalization, which they failed to do. The AEM loss function even led to a significant decrease in performance on the test set. Hence, we consider the simpler basic approach as the favored choice.

**Table 5:** Results from fitting Cox proportional hazards models on the results from the external data. Because we only have one predicted probability for each patient here, we use a time-independent model. The hazard ratio is the change in hazard when the estimated probability is increased by 0.1. We also present the C-index and the p values following Wald’s test.

Model variations	Hazard ratio	C-index	Wald’s test (p)
Basic	1.30 (1.17 to 1.45)	0.699	<0.001^a^
SKS	1.30 (1.15 to 1.48)	0.676	<0.001^a^
AEM	1.18 (1.05 to 1.33)	0.612	0.006^a^
GG	1.45 (1.21 to 1.73)	0.679	<0.001^a^

a Indicates statistical significance with a p value below 0.05.

When selecting operating points for our models, we again see difficulties in generalizability. In addition to large drops in accuracy for all models when evaluated on the external dataset, the large differences between sensitivity and specificity for the models indicate that these operating points are not optimal. For these models to be considered for clinical use, the robustness and stability of the operating point need to be substantially improved. Given that we used the maximum Youden’s J statistic as a criterion for selecting these thresholds, which should provide an even balance between sensitivity and specificity, and still did not achieve this, we consider our models as not yet clinically usable.

The basic model’s predictions on the test set gave the best fit of the time-dependent Cox proportional hazards model, demonstrating a clear association with the longitudinal patient outcomes. The GG model did not show any significant effect of the covariate, with the confidence interval of the hazard ratio overlapping 1 and a p value above 0.05 for the Wald’s test. This further supports the claim that benign tissue contains important prognostic information and that longitudinal predictions in prostate cancer patients should not rely solely on Gleason grades. Interestingly, all models showed significant effects for the time-independent Cox model on the external data. However, this model is simpler than the time-dependent model, and because we have a binary outcome, the C-index becomes closely related to the AUROC. The survival predictions could be improved with a model trained towards a survival analysis-oriented objective. More advanced deep learning techniques such as generative adversarial networks (GANs)^46^ and graph convolutional neural networks (GCNs)^47^ have been suggested in the literature for survival analysis in computational pathology. Employing these techniques could be beneficial; however, they will likely require a larger dataset. We leave this investigation as future work.

There are currently no standardized screening programs for prostate cancer, despite its current high (and rising) prevalence. The use of PSA screening in a clinical setting has faced resistance due to concerns of over-treatment, as demonstrated by the ERSPC trials.^19^ Despite them proving a reduction in mortality, in 2012, the US Preventive Services Task Force issued a recommendation against PSA screening.^7^ However, active surveillance of patients with elevated PSA levels has been suggested as a viable alternative to radical treatment, which can mitigate the risk of over-treatment.^6^^,^^8^ With the inclusion of MRI and more advanced blood tests in the diagnostic procedure, this can be improved even further and may make prostate cancer screening programs feasible.^4^^,^^20^^,^^22^ We argue that an AI-based solution, capable of providing reliable longitudinal predictions of the monitored patients in this setting, could be highly beneficial. It holds the potential of both assisting the pathologists in their work, reducing their increasing workload, as well as improving the diagnostic accuracy by catching high-risk patients at an earlier stage.

## 5 Conclusion

In this work, we have presented an ABMIL-based approach for predicting longitudinal outcomes of prostate cancer patients on active surveillance. Our models take only H&E-stained WSIs as input, following a patient visit, and predict whether this patient should remain on active surveillance or receive treatment for prostate cancer. We do this by learning on patient-level labels, without using any explicit Gleason grades, hence moving toward a diagnostic tool that is independent of the variability of the grading system. In concordance with previous studies, our results suggest that the prostate tissues with ISUP grades of cancer do not hold all relevant prognostic information. By studying benign tissue, some of these prognostic factors can be identified, improving longitudinal predictions. We extend our previous work^43^ by including longitudinal predictions up to 30 months into the future, and we demonstrate strong predictions with an AUC of 0.958 (95% CI, 0.957 to 0.959) and significant predictive capabilities with the fitted Cox models. However, none of our models generalized to the external dataset. More work, both in terms of data collection and algorithm development, is needed before these models can provide any clinical value. Yet, this study demonstrates that it is possible to develop an AI solution for monitoring prostate cancer patients on active surveillance and detecting high-risk patients earlier than with traditional diagnostic methods.

## Data Availability

Our code for training and evaluating our models will be publicly available at https://github.com/fwinzell/PRIAS_outcome_prediction. The external dataset is openly accessible at the Swedish National Data Service.^39^ Our dataset cannot be shared due to the current ethical approval of the PRIAS study.
